# Maternal Inheritance of U’s Triangle and Evolutionary Process of *Brassica* Mitochondrial Genomes

**DOI:** 10.3389/fpls.2020.00805

**Published:** 2020-06-12

**Authors:** Jia-Yu Xue, Yue Wang, Min Chen, Shanshan Dong, Zhu-Qing Shao, Yang Liu

**Affiliations:** ^1^Center for Plant Diversity and Systematics, Institute of Botany, Jiangsu Province and Chinese Academy of Sciences, Nanjing, China; ^2^College of Horticulture, Nanjing Agricultural University, Nanjing, China; ^3^Fairy Lake Botanical Garden, Shenzhen & Chinese Academy of Sciences, Shenzhen, China; ^4^State Key Laboratory of Pharmaceutical Biotechnology, School of Life Sciences, Nanjing University, Nanjing, China

**Keywords:** mitochondrial genome, phylogenomics, paleogenomics, *Brassica*, maternal inheritance, evolutionary history

## Abstract

The sequences and genomic structures of plant mitochondrial (mt) genomes provide unique material for phylogenetic studies. The nature of uniparental inheritance renders an advantage when utilizing mt genomes for determining the parental sources of hybridized taxa. In this study, a concatenated matrix of mt genes was used to infer the phylogenetic relationships of six cultivated *Brassica* taxa and explore the maternal origins of three allotetraploids. The well-resolved sister relationships between two pairs of diploid and allotetraploid taxa suggest that *Brassica carinata* (*car*) possessed a maternal origin from *Brassica nigra*, while *Brassica juncea* (*jun*) was maternally derived from *Brassica rapa* (*cam*). Another allotetraploid taxon, *Brassica napus* (cv. Wester) may have been maternally derived from the common ancestor of *B. rapa* and *Brassica oleracea* (*ole*), and/or have undergone (an) extra hybridization event(s) along its evolutionary history. The characteristics of *Brassica* mt genomic structures also supported the phylogenetic results. *Sinapis arvensis* was nested inside the *Brassica* species, sister to the *B. nigra–B. carinata* lineage, and possessed an mt genome structure that mostly resembled *B. nigra*. Collectively, the evidence supported a systematic revision that placed *S. arvensis* within *Brassica.* Finally, ancestral mt genomes at each evolutionary node of *Brassica* were reconstructed, and the detailed and dynamic evolution of *Brassica* mt genomes was successfully reproduced. The mt genome of *B. nigra* structurally resembled that of the *Brassica* ancestor the most, with only one reversion of a block, and the *Brassica oleracea* underwent the most drastic changes. These findings suggested that repeat-mediated recombinations were largely responsible for the observed structural variations in the evolutionary history of *Brassica* mt genomes.

## Introduction

Green plants possess three independent genetic systems that are encoded by the genomes in nuclei, chloroplasts, and mitochondrion. Of the three genomes, the nuclear (nu) genome is biparentally inherited between generations, while the chloroplast (cp) and mitochondrial (mt) genomes, referred to as organellar genomes, are uniparentally inherited. Specifically, plant organellar genomes are overwhelmingly maternally inherited, except in some gymnosperm lineages where these genomes are paternally inherited, including Pinaceae, Cupressaceae, and Taxodiaceae (Mogensen, 1996; Jansen and Ruhlman, 2012; Worth et al., 2014). Distinct evolutionary histories between the nu and organellar genomes occasionally lead to incongruent phylogenetic results (Qiu et al., 2006; Jansen et al., 2007; Moore et al., 2007; Qiu et al., 2010; Li et al., 2019; One Thousand Plant Transcriptomes Initiative, 2019) which could identify the underlying hybridization events hidden within evolutionary history (Bastide et al., 2018; Lecaudey et al., 2018). Therefore, uniparental-origin characteristics render a natural advantage in the parental identification of organellar genomes. Compared with cp genomes, plant mt genomes have a more dynamically organized genomic structure in terms of gene content and order, which could be affected by block reversions and translocation events, gene (intron) gains and losses, alien DNA insertions, and pseudogenization, and could be used as alternative evidence for inferring evolutionary histories (Li et al., 2009; Wang et al., 2009; Xue et al., 2010; Liu et al., 2012a, b; Dong et al., 2018a, b). In contrast, the cp genome has a much more conserved structure and cannot be applied for such analyses (Jansen and Ruhlman, 2012).

**FIGURE 1:**
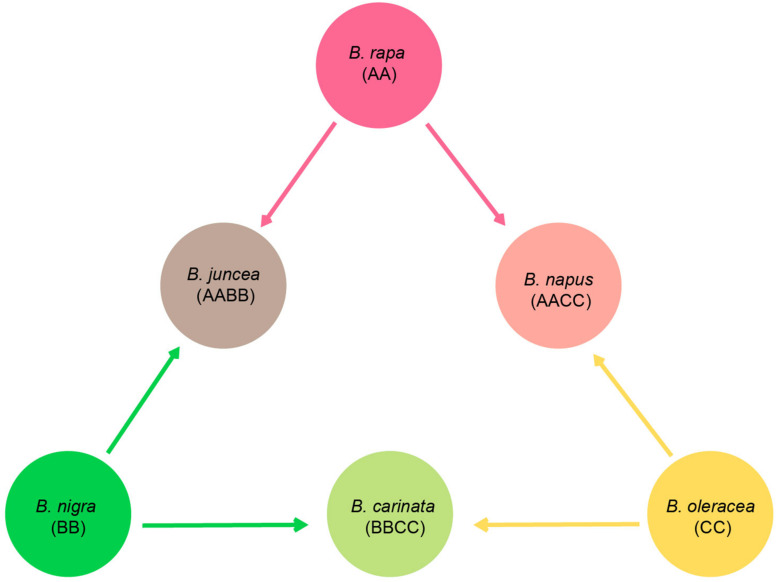
Diagram of U’s triangle showing the genetic relationships among six cultivated *Brassica* taxa (Nagaharu, 1935).

Brassicaceae is comprised of over 330 genera and 3,800 species that are distributed worldwide (Bailey et al., 2006; Huang et al., 2016). Among the genera, *Brassica* is the most important genus as it is comprised of many important vegetables and oil crops (e.g., cabbage, broccoli, cauliflower, kale, and rapeseed). These economically important plants almost all belong to six cultivated species, *Brassica rapa*, *Brassica juncea*, *Brassica nigra*, *Brassica carinata*, *Brassica oleracea*, and *Brassica napus*. Based on artificial inter-specific hybridization experiments, a well-known model, U’s triangle, was proposed to demonstrate the genetic relationships among these six species (Figure 1; Nagaharu, 1935). *B. rapa* (AA, 2n = 2x = 20), *B. nigra* (BB, 2n = 2x = 16), and *B. oleracea* (CC, 2n = 2x = 18) are three basic diploid species, and through natural hybridization and genome doubling, three allotetraploid species were derived, including *B. juncea* (AABB, 2n = 4x = 36), *B. carinata* (BBCC, 2n = 4x = 34), and *B. napus* (AACC, 2n = 4x = 38). This hypothesis has been increasingly accepted as the nu genomes of *Brassica* taxa have been successfully sequenced. Comparative genomic analyses can assign the subgenomes of the allotetraploids, *B. juncea* and *B. napus*, with their diploid parental taxa, and the results were in agreement with U’s triangle (Chalhoub et al., 2014; Yang et al., 2016a). Along with the supporting evidence from nu genomic comparisons, the genetic relationships of U’s triangle taxa are becoming a mainstream theory, while much of the evolutionary history among these species remains ambiguous, including the identification of particular paternal and maternal sources of each hybridized taxon and the detailed processes.

Organellar genomes have long been used as materials to address evolutionary questions regarding *Brassica* taxa, especially the three allotetraploid species. Previous studies using cp and mt genomic data overwhelmingly support the hypothesis that the maternal source of *B. juncea* comes from *B. rapa* (Li et al., 2017; Kim et al., 2018) but two studies based on a large number of *B. juncea* varieties, discovered multiple origins of the allotetraploid (Chen et al., 2013) and a few varieties with a maternal origin of *B. nigra* (Kaur et al., 2014). In fact, *B. nigra* has been suggested to be the maternal parent of another allotetraploid *B. carinata* (Yamagishi et al., 2014; Yang et al., 2016b; Li et al., 2017) but it’s not known if there is an undiscovered *B. carinata* variety with a maternal parent of *B. oleracea*. A more complicated scenario arises regarding the other allotetraploid, *B. napus*. In a study based on cp genomes, several *Brassica* morphotypes were analyzed. The results revealed that *B. napus* always clustered with *B. rapa* morphotypes, and thus, it was postulated that the maternal origin of *B. napus* was *B. rapa*. However, as *B. rapa* and *B. napus* morphotypes did not cluster into a monophyletic group, but were distantly separated by *B. juncea* and *B. oleracea*, the independent origins of *B. rapa* and *B. napus* were hypothesized, including complicated evolutionary processes involving extra hybridization events with wild taxa and subsequent continued back-crossings under natural conditions (Li et al., 2017). Although such convergent evolution of different morphotypes in East Asia and Europe sounds much too coincidental, multiple hybridization events throughout *B. napus* evolution were proposed in previous studies (Palmer et al., 1983; Allender and King, 2010). In another study that also adopted cp genomes, all *B. napus* clustered, being sister to the clade comprising *B. rapa*, *B. juncea*, and *B. oleracea* (Kim et al., 2018). An et al. (2019) collected 183 *B. napus* accessions, and the cp phylogenomic analysis indicated that some *B. napus* accessions clustered in the *B. rapa* clade or *B. oleracea* clade and vice versa. The results from mt genomes analyses are largely in accordance with that from cp genomes, which suggest that *B. napus* has two mitotypes, represented by *pol* and *nap* (Chen et al., 2013). Among the two mitotypes, *pol* should be maternally derived from *B. rapa*, indicated by the phylogeny and the genomic structures (Chang et al., 2011; Heng et al., 2017) but the other mitotype *nap* is phylogenetically sister to a clade comprising *B. rapa*, *B. juncea*, and *B. oleracea*, implying a more complicated evolutionary history and an unidentified maternal parent (Chang et al., 2011; Yang et al., 2016b).

Despite many efforts devoted to determining the maternal and paternal sources of the three hybridized *Brassica* species, their evolutionary origins seem to remain unclear. In this study, an mt phylogenomics approach was adopted to reconstruct the phylogeny of six *Brassica* taxa and determine the maternal parents of the allotetraploids. The results were mutually supported by the mt genomic structural characteristics. Furthermore, a paleogenomic algorithm was developed to reconstruct the ancestral mt genomes. Using this method, a detailed maternal evolutionary history of U’s triangle was presented for these *Brassica* species.

## Results

### Mt Phylogeny Presents Maternal Inheritance of *Brassica* Taxa

A concatenated matrix of 30 protein-coding genes from 12 Brassicales mt genomes were assembled and subsequently used for mt phylogenetic reconstruction (Supplementary Table S1). Among the sampled taxa, 10 belonged to *Brassicaceae* and two belonged to *Bataceae* and *Caricaceae*. Other *Brassica* taxa were used as the outgroups and references for the assessment of the phylogeny of U’s triangle taxa. After removing ambiguous positions, the concatenated nucleotide dataset comprised of 27,612 characters with 1,485 variable sites and 602 parsimony-informative sites.

**FIGURE 2:**
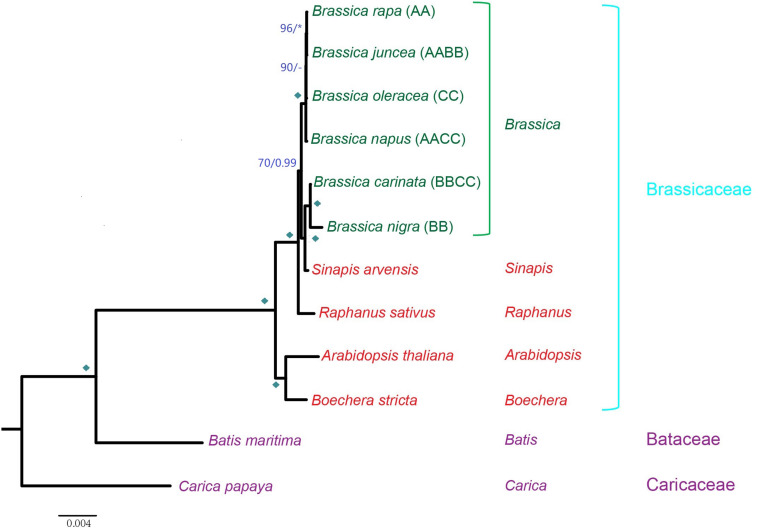
Phylogenetic tree inferred from concatenated nucleotide sequences of all mt protein-coding genes. ML BS support values and Bayesian posterior probabilities are labeled. Diamonds indicate both BS of 100% and PP of 1.00. Asterisks indicate either BS of 100% or PP of 1.00. BS support values <50% or Bayesian posterior probabilities <0.5 are indicated as “–”.

The concatenated matrix yielded a phylogeny as follows: *B. nigra* is sister to *B. carinata* (*car*) and composed of a monophyletic group with *Sinapis arvensis*, and this clade is sister to the other comprising four *Brassica* taxa. Among the four taxa, *B. napus* (cv. Wester) shows the earliest divergence, successively followed by *B. oleracea* (*ole*), *B. rapa* (*cam*), and *B. juncea* (*jun*) (Figure 2). Since all of the allotetraploids are supposed to cluster with their maternal parents, it is clear that *B. juncea* has a maternal origin of *B. rapa*, and the maternal origin of *B. carinata* is *B. nigra*. The other allotetraploid, *B. napus*, was a recovered sister to the clade comprising *B. oleracea*, *B. rapa*, and *B. juncea*. Based on the phylogenetic position of *B. napus*, its maternal origin should be theoretically derived from the common ancestor of the three taxa, which was likely a wild *Brassica* species. However, the possibility that the *B. napus* mt genome has undergone a distinct evolutionary route due to particular selective pressures after the formation of this taxon could not be ruled out or may involve additional hybridization events. Interestingly, *S. arvensis* was recovered inside the *Brassica* genus, sister to the *B. nigra* and *B. carinata* lineage. A short evolutionary distance among the *Brassica* taxa can be observed, especially among *B. rapa*, *B. juncea*, *B. oleracea*, and *B. napus*. These close evolutionary relationships provide genetic possibilities for frequent hybridization events in this genus.

### Mt Genomic Structure Analysis Supports Mt Phylogenomic Inferences

**FIGURE 3:**
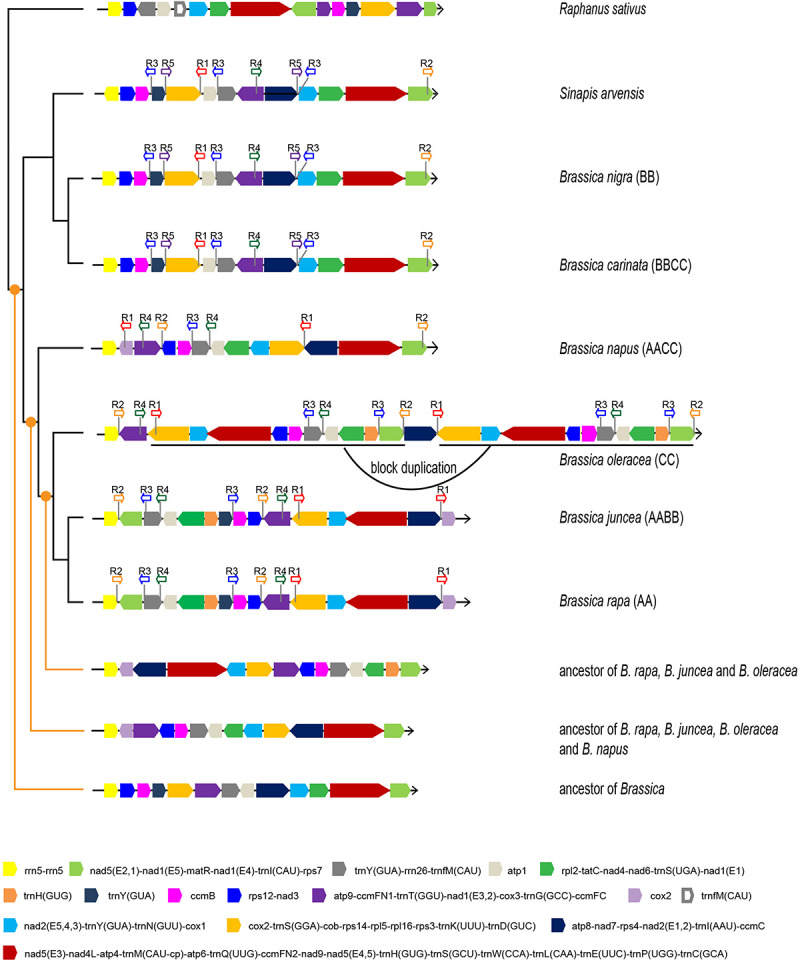
Mt genomic structures of six *Brassica* taxa, *S. arvensis*, and *R. sativus*. Different basic syntenic blocks are presented in different colors. Arrows indicate block directions. The locations and directions of repeat sequences are labeled. The inferred ancestral mt genomic structures are presented at three different evolutionary nodes.

Apart from gene sequences, mt genomic organization is an effective resource for studying the evolutionary relationships among taxa. Since the mt phylogeny did not fully identify the maternal origins of all allotetraploids, the mt genomic structure characteristics were used as alternative evidence. The mt genomic organization of six *Brassica* taxa and two close relatives (*S. arvensis* and *Raphanus sativus*) were analyzed and compared. All of the mt genomes were artificially aligned in linear forms, starting with the rRNA gene *rrn5* (Figure 3 and Supplementary Table S2).

The analysis recognized 15 basic syntenic blocks in the *Brassica* taxa, among which five contain a single gene, and the rest comprised of at least two genes. Consistent with the phylogenetic results, two pairs of taxa (*B. rapa* and *B. juncea*, and *B. nigra* and *B. carinata*) exhibit identical mt genomic organizations, indicating the maternal origins of *B. juncea* and *B. carinata*. The other taxa showed a structural disagreement with one another. *B. oleracea* experienced a 114-kb block duplication and it was thus inferred that the duplication is species-specific and should have occurred recently as almost no sequence divergence was observed between the duplicated blocks and the duplication was not shared with other taxa. The synteny of multiple basic blocks was observed between *B. napus* and other taxa, and the mt genome of *B. napus* resembled that of *B. oleracea* and *B. rapa* more than other taxa. *B. napus* shared a two two-block synteny with *B. rapa* (purple-blue and light gray-green) and shared a two two-block (light green-yellow and orange-blue black) and one four-block (blue-pink-gray-light gray) synteny with *B. oleracea*; these three taxa shared one two-block synteny (light blue-orange). Therefore, the result of mt genomic structure analysis agrees with the inference from the phylogenomic analyses that *B. napus* is likely to be maternally derived from the common ancestor of *B. oleracea* and *B. rapa*. Unless more new wild *Brassica* mt genomes are sequenced, these inferences remain to be verified.

*Sinapis arvensis*, the suspected systematically false-classified taxon, exhibited the exact same mt genomic structure as *B. nigra* and *B. carinata*, except the direction of one basic block (yellow) (Figure 3). Clearly, the results based on mt genomic organization agreed with the phylogenetic results based on gene sequences, which strengthen the reliability of the phylogenetic results.

### Tracing the Maternal Evolution of Mt Genomes by Paleogenomic Reconstruction

With the well-resolved phylogeny and a newly developed algorithm, the mt genome structures of *Brassica* ancestors were inferred and used to reconstruct a detailed evolutionary process of *Brassica* mt genomes.

**FIGURE 4:**
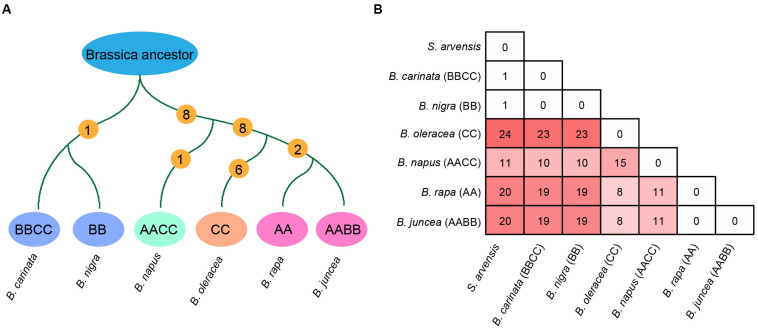
**(A)** Steps of structural changes from the *Brassica* common ancestor to each extant *Brassica* taxa. **(B)** Number of structural changes between all *Brassica* taxa and *S. arvensis*.

Firstly, the ancestral mt genomes at three different evolutionary nodes were reconstructed in this study (Figure 3). The common ancestor of all *Brassica* was inferred to have an mt genome resembling the early diverging *B. nigra* lineage. Although 15 basic blocks were identified in *Brassica*, the ancestor had only 13, and the other two were acquired later during evolution. From the parsimonious perspective, since the divergence with the common ancestor of *Brassica*, *B. nigra* has experienced only one reversion (dark gray-gray-purple), which could bring it absolutely collinear with the *Brassica* ancestor (Figure 4A). Differing from the *B. nigra* lineage, the other taxa seemed to experience more drastic genomic rearrangements. The common ancestor of *B. napus*, *B. oleracea*, *B. rapa*, and *B. juncea* underwent eight changes, including six reversions and two gene gains [*cox1* and *trnY(GUA)*] by duplication after splitting from the *B. nigra* lineage. Afterward, since the rise of *B. napus*, its mt genome has remained conservative and stayed collinear with its ancestor, except losing a copy of the duplicated gene *trnY(GUA)*, while the other three taxa continued their genomic arrangements. *B. oleracea* underwent five reversions, two gene gains, and one gene loss before divergence from the *B. rapa* lineage; and afterward, three reversions, two gene losses, and one duplication of the 114 kb block formed its current structure (a total of 22 changes since the divergence with the *Brassica* ancestor). Finally, the *B. rapa* lineage experienced two recent reversions and a total of 18 changes. With this algorithm, the structural evolution of all of the *Brassica* mt genomes was successfully inferred. Collectively, the six taxa have undergone 26 genomic changes, since the divergence of their last common ancestor. Additionally, the steps needed to bring any two mt genomes to be collinear with one another were also calculated (Figure 4B). For example, 11 steps were needed to make *B. rapa* and *B. napus* completely collinear, but 23 steps were needed to make *B. oleracea* and *B. nigra* collinear.

### Rearrangement and Repeats

Repeat regions are considered to be closely associated with mt genomic rearrangements, causing reversions and translocations. Therefore, in order to test the reliability of inferred structural variations, repeat sequences (no shorter than 50 bp) in each of these *Brassica* mt genomes were searched (Supplementary Table S3). Then, comparative analyses were performed to determine the homologous relationships among different taxa and identify their locations in the mt genomes associated with specific blocks. These repeats were mainly identified within inter-genic spaces, of which, some contain partial gene sequences.

The search identified five different repeat regions (sequence length ≥200 bp in at least one taxon) and was shared by at least three taxa (Figure 3). Among the identified repeats, sequence lengths and copy numbers varied among different taxa. Through a BLAST search, their homology was easily recognized. For example, the repeat R3 in *B. rapa* has a length of 228 bp, but in *B. nigra* and *B. oleracea*, the lengths are 232 and 257 bp, respectively. *B. oleracea* and the *B. nigra* lineages have three copies of R3s, while the *B. rapa* lineage has only two copies and *B. napus* lineage has only one copy. Coincidently, these repeats are all located at the boundaries of previously recognized basic blocks, suggesting a close association with the repeat and rearrangement events.

## Discussion

### Mt Phylogenomics and Structural Characteristics Can Help to Reveal Maternal Inheritance and Evolution

Both mt gene sequences and structures are considered to be good materials for evolutionary studies, especially in phylogenetic and comparative genomics studies (Qiu et al., 2006, 2010; Xue et al., 2010; Liu et al., 2012a, b). The uniparental inheritance nature of the mt genome renders an extra function for distinguishing parental origins of hybridized taxa, recognizing the donor of mt. Although the sequence of cp genomes theoretically plays an equal important role, additional evidence from mt genomic structures could be used to verify such results or provide an alternative hypothesis.

In this study, the results derived from *Brassica* mt genomes largely agree with those of previous studies based on cp or mt genomes (Palmer et al., 1983; Allender and King, 2010; Yang et al., 2016b; Li et al., 2017; Kim et al., 2018) from which the maternal origins of two allotetraploids, *B. juncea* (*jun*) and *B. carinata* (*car*), were determined. Nevertheless, with regard to the maternal origin of *B. napus* (cv. Wester), some ambiguity remains. The phylogenetic analysis of this study placed *B. napus* as a sister to the clade comprising *B. rapa* (*cam*), *B. juncea*, and *B. oleracea* (*ole*). These evolutionary relationships are in accordance with our analysis toward mt genomic structural characteristics as *B. napus* exhibited structural features shared by both *B. rapa* and *B. oleracea* (Figure 3). Thus, subsequent hybridization events in *B. napus* appear to have occurred rather than species-specific variations without hybridization. As mt sequence variations and genomic structural variations underwent parallel routes of evolution, the convergent results are easily explained by another single mt donor, which appears likely to be the ancestor of *B. rapa*, *B. juncea*, and *B. oleracea*. However, whether one or multiple hybridization events occurred during the evolutionary history of *Brassica* could not be inferred as only one morphotype of *B. napus* was analyzed in this study. In future studies, more morphotypes should be analyzed to confirm these findings.

*Brassica nigra* and *B. carinata* are resolved as an early diverging lineage in *Brassica*, which are distantly related to other *Brassica* taxa, thereby explaining their lack of applicability in the artificial hybridization of new taxa with higher economic values. *S. arvensis* belongs to another genus *Sinapis* genus, but is phylogenetically recovered within *Brassica*, sister to the *B. nigra* lineage. Such a close relationship between *Sinapis* and *B. nigra* is not rarely seen in other studies. Other than our result, two studies using cp markers also recovered similar relationships (Warwick and Black, 1991; Arias and Pires, 2012). Interestingly, the mt genome structure of *S. arvensis* is almost absolutely collinear to that of the *B. nigra* lineage (Sang et al., 2020) except one block reversion and matches its position in the phylogenetic tree. The reason for the close relationship of *S. arvensis* and *B. nigra*, as we infer, can be explained by two hypotheses: one is that *S. arvensis* is truly an evolutionary close branch to *B. nigra*; the other may involve a hybridization event of *S. arvensis*, with *B. nigra* being the maternal parent. However, the nuclear chromosome number of *S. arvensis* (2n = 18) indicates that it is a diploid species that did not undergo hybridization events in its evolutionary history (Arumuganathan and Earle, 1991). Morphologically, *S. arvensis* resembles *Brassica* taxa in many characteristics, including simple and lyrate-pinnatifid leaves, yellow petals, oblong to linear heteroarthrocarpous siliques, and uniseriate seeds. The one obvious difference separating the *Brassica* and *Sinapis* genera is that the *Brassica* taxa have a prominent midvein, while *Sinapis* possess 3–5 veins. Additionally, there is a large overlap between the geographic distributions of these two species (Scholz, 1919; Cheo et al., 2001). Therefore, the phylogenetic results suggest that *S. arvensis* be placed within the *Brassica* genus; its mt genomic organization and morphological characteristics with *B. nigra* strongly support this new systematic classification. Because *S. arvensis* is not the outermost lineage in the phylogenetic tree, but is nested within a clade comprising *B. nigra* and *B. carinata*, the results suggest that *Sinapis* should be merged with *Brassica*. Yet, the taxonomic reassessment regarding the position of *Sinapis* requires additional data from nuclear genomes and more *Sinapis* species. Actually apart from *Sinapis*, species from other genera are also commonly placed into *Brassica* through phylogenetic reconstructions, like the radish (*Raphanus sativus*) by both cp and nuclear data (Palmer et al., 1983; Huang et al., 2016; Kim et al., 2018). But in mechanism the radish might be different from *Sinapis*, because our mt phylogenomic result does not support placing the radish inside *Brassica*, and the morphological differences between the radish and *Brassica* species are quite distinguishable. Therefore, cytoplasmic cp DNA transfer between species via introgression sounds more likely for radishes. To sum up, the non-monophyly of *Brassica* may attribute to the false systematic classification and high potential of hybridization among Brassicaceae taxa.

### Step-by-Step Tracing of a Detailed and Dynamic Evolutionary History Based on an Ancestral Genome Reconstruction Approach

Evolution refers to a dynamic process spanning a specific period of time. Focusing on the comparison of extant taxa only obverse current similarities and differences, specific evolutionary events can be investigated and our understanding of the mechanisms that gave rise to their current status can be enhanced. This study introduces the concept of mt paleogenomics, which utilizes putative ancestral mt genomes in evolutionary studies. The gene-based reconstruction approach recognizes basic syntenic blocks shared by all surveyed taxa, and these blocks are subsequently used as elements for ancestral structure reconstruction. The ancestral mt genomes are determined by the collinearity of basic blocks between the phylogenetic in-group taxa and outgroup, or earliest-diverging taxa. Moreover, this strategy requires a well-resolved phylogeny. With this ancestral reconstruction approach, ancestral mt genomes of progenitors at multiple evolution nodes were inferred in this study.

The paleogenomics strategy has been applied to the reconstruction of ancestral nu genomes in several plant lineages (Murat et al., 2015, 2017). However, detailed evolutionary processes could not be correspondingly reproduced as nu genomes are too large, and their evolution involves many complicated events. mt genomes are much simpler than nu genomes, but more complicated than cp genomes due to a diversity of structural variations resulting from block reversions, translocations, *trans-*spliced introns, gene (intron) losses and gains, horizontal gene transfers, and alien DNA insertions. The moderate complexity of the mt genome renders its practicability for further investigating detailed events over evolutionary history and uncovering the associated underlying mechanisms.

It should be noted that successful paleogenomic reconstruction requires the adequate sampling of taxa with sequenced genomes, including properly selected outgroups and offspring lineages. A shortage of taxon-sampling may lead to a failed reconstruction of, if not all, a part of ancestral genomic regions. Fortunately, with the rapid development of sequencing technology, there will be more applications of this strategy in the future.

### Repeat-Mediated Recombinations Cause Block Reversions and Lead to Complicated Rearrangements

Among the factors influencing mt genome organization, reversions by repeat sequences play a dominant role in the diversity of plant lineages from early land plants bryophytes and lycophytes to relatively late-diverging gymnosperms and angiosperms. Of the 26 inferred structural variations in *Brassica* mt genomes, the majority (17) belonged to repeat-mediated reversions, almost two times as the total number of other events (9), which included four gene gains, four gene losses, and one block duplication.

**FIGURE 5:**
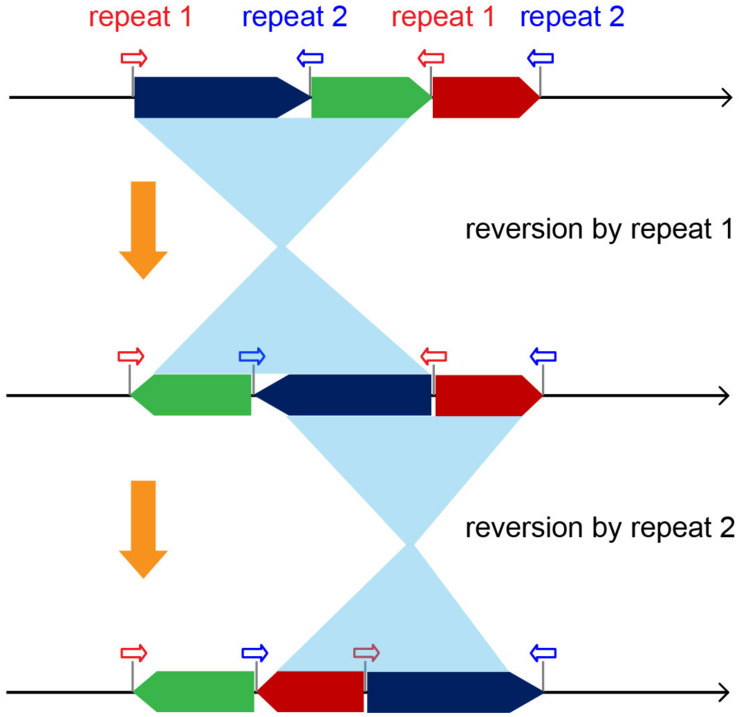
Diagram demonstrating the complicated mt genomic rearrangements caused by the repeat-driving reversions.

In theory, inverted repeats could lead to a reversion of a genomic region located in between (Xue et al., 2010; Liu et al., 2012a). Thus, multiple groups of repeats scattered over mt genomes could give rise to complicated rearrangements and result in completely different genomic structures (Figure 5). Five groups of repeats with lengths ≥200 bp were recognized and shared by all of the *Brassica* mt genomes, and their locations were identified at the boundaries of basic blocks, suggesting a close association with a diversity of rearrangement events. Specifically, each *Brassica* taxa had a minimum of 23 pairs of repeats (length ≥50 bp, Supplementary Table S3) in their mt genomes, all of which were potential materials for triggering recombination, implying that more genomic changes had occurred in their evolutionary histories or could occur in the future. The repeat-mediated reversion is an early rising mechanism in plant mt genomes, dating back to ∼4.8 million years ago, the origin of land plants (Wang et al., 2009; Xue et al., 2010; Liu et al., 2011). In early land plants, such as mosses, reversions have remained relatively silent, but since the emergence of vascular plants, mt genomes have been more and more active with reversions (Li et al., 2009; Liu et al., 2011, 2012a). Thus, in this study, it is hypothesized that with the expansion of plant mt genomes, more repeats are generated, causing an increase in genomic plasticity. A particular example verifies this notion. Specifically, the mt genomes of mosses lack repeat sequences and have completely identical structures among different taxa, indicating extreme conservation and stability within this lineage (Liu et al., 2011, 2014).

Angiosperms possess diverse mt genomic structures that were largely driven by repeat sequences. However, different repeats originated at different evolutionary nodes. For example, the *Brassica* R1 repeat is widely found in a number of rosids, one asterid taxon, and in the basal angiosperm *Amborella*. This repeat likely has an early angiosperm origin, but has been independently lost in some lineages, such as monocots and most asterids. The R2 repeat likely has a eudicot origin with homologs found in a wide range of eudicot lineages, but none in other angiosperm lineages. Additionally, some repeats are species-specific or limited to only a few close-related taxa, such as the 114 kb repeats in *B. oleracea* and 6.5 kb repeats in *B. nigra* and *B. carinata*. Therefore, these repeats are believed to have been recently derived. With such diverse repeat sequences, it is clear that repeat-mediated reversions have become a major force that has shaped plant mt genomes over time.

## Materials and Methods

### Data Sources and Processing

The 12 available mt genomes of Brassicales taxa were downloaded from the National Center for Biotechnology Information (NCBI) database. Voucher information and GenBank accession numbers of the analyzed samples are provided in Supplementary Table S1. These mt genomes were initially annotated by different research groups with different methods and styles of nomenclature, and these annotations were revised and the nomenclature was unified to facilitate next-stage data processing and analyses. Pseudogenes refer to genes that have lost functions and can be distinguished as follows: (1) the loss of a fragment of coding sequence; and (2) insertions, deletions, and mutations of nucleotides breaking the open reading frames. First, OGDRAW was used for automated annotation (Greiner et al., 2019). Then, the results of the two versions were compared. The discrepancy between the two annotated versions was manually examined to determine the final version.

### Phylogenetic Analysis

Mt protein-coding genes were individually aligned using MAFFT to build amino acid alignments (Katoh et al., 2005). In all cases, poorly aligned regions were trimmed using GBLOCKS with the least stringent settings (Talavera and Castresana, 2007); nucleotide alignments were produced based on corresponding amino acid alignments after the removal of ambiguous positions. The above processes were automatically conducted using TranslatorX (Abascal et al., 2010). After removing stop codons, the 30 single-gene nucleotide alignments were concatenated into final alignments and converted into appropriate formats using Geneious v6.0.3 (Biomatters, New Zealand).

Collectively, 30 mt protein-coding genes shared by the six *Brassica* species were used for the phylogenetic analyses; pseudo and missing genes were treated as gaps in the analyses. The concatenated nucleotide dataset was analyzed by the maximum-likelihood (ML) method and Bayesian inference (BI). ML analyses were performed using the parallel version of RAxML v7.2.3 (Stamatakis, 2006). Bayesian analyses were inferred by Mr. Bayes using the GTR+G model with two runs of four chains (Ronquist et al., 2012). Posterior probabilities (PPs) of clade support were estimated by sampling trees from the posterior distribution after removal of the burn-in samples. Nonparametric bootstrap (BS) analyses were implemented by GTR+CAT approximation of 100 pseudoreplicates. PartitionFinder was used for selecting optimal data partition schemes and associated substitution models (Lanfear et al., 2012). Using prior gene regions and codon positions, seven partitions were selected as the best scheme for nucleotide data. Moreover, using prior gene regions, four partitions were selected as the best partitioning scheme. The optimal partitioning scheme was then used in subsequent phylogenetic analyses. *Carica papaya* was used as the outgroup.

### Mt Paleogenomic Reconstruction

The order of mt genes (exons separated by other genes due to *trans-*spliced introns were considered independent genes) of each taxon was extracted from the corresponding mt genome annotations. The rRNA gene *rrn5* was selected as the starting point, and all of the genes were aligned accordingly. Gene directions were also considered. The gene-based paleogenomics approach first identified syntenic blocks shared by all surveyed taxa, and these blocks were used as basic units for subsequent ancestral structure reconstruction. This strategy required a well-resolved phylogeny prior to subsequent analyses.

Based on a recovered phylogeny, the determination of ancestral mt genomic structures followed specific algorithms. For the lineage derived from a common ancestor, the collinearity of basic blocks between any interior taxon of the lineage and the outgroup (Supplementary Figure S1, case 1), as well as the collinearity of basic blocks between one taxa in the earliest-diverging branch and others in later-diverging (interior) branches (Supplementary Figure S1, case 2), was considered the ancestral genomic structure. With this ancestor reconstruction approach, the ancestral mt genomes of progenitors were inferred at multiple evolution nodes.

### Repeat Identification and Analysis

All of the repeat regions (≥50 bp) and their relative directions were searched for in each mt genome using the REPuter software (Kurtz et al., 2001). Homology of the repeats was determined following a basic local alignment search tool (BLAST) search of the non-redundant (Nr) NCBI database (Altschul et al., 1990).

## Data Availability Statement

Publicly available datasets were analyzed in this study. The data can be found here: NC_012116, NC_024429, NC_037304, NC_008285, NC_016120, NC_016123, NC_016118, NC_016125, NC_031896, NC_029182, and NC_018551.

## Author Contributions

J-YX and YL designed the study. J-YX, YW, MC, YL, SD, and Z-QS analyzed the data. J-YX wrote the manuscript. Z-QS, YW, MC, and YL participated in the revision of the manuscript. All authors read and approved the final manuscript.

## Conflict of Interest

The authors declare that the research was conducted in the absence of any commercial or financial relationships that could be construed as a potential conflict of interest.

## Supplementary Material

FIGURE S1 Algorithm of ancestral structure determination.

TABLE S1 List of 12 Brassicales taxa with the mitochondrial genomes used in this study.

TABLE S2 Gene composition and order of 12 Brassicales mt genomes. Genes in red are miss-annotated in previous annotation. “+” and “−” indicate the position of genes in the standard chain and complimentary chain, respectively.

TABLE S3 List of repeats detected in the mitochondrial genomes of six *Brassica* taxa and *S. arvensis*. “F” indicates relatively forward direction between repeats; “I” indicates relatively inverted direction between repeats. The starting positions of repeats are recorded in the columns of copy number.
