# Supplementary figures and images for: Maternal Inheritance of U’s Triangle and Evolutionary Process of Brassica Mitochondrial Genomes
Source: Front Plant Sci. 2020 Jun 12;11:805. doi: 10.3389/fpls.2020.00805 (PMC7303332; doi:10.3389/fpls.2020.00805)

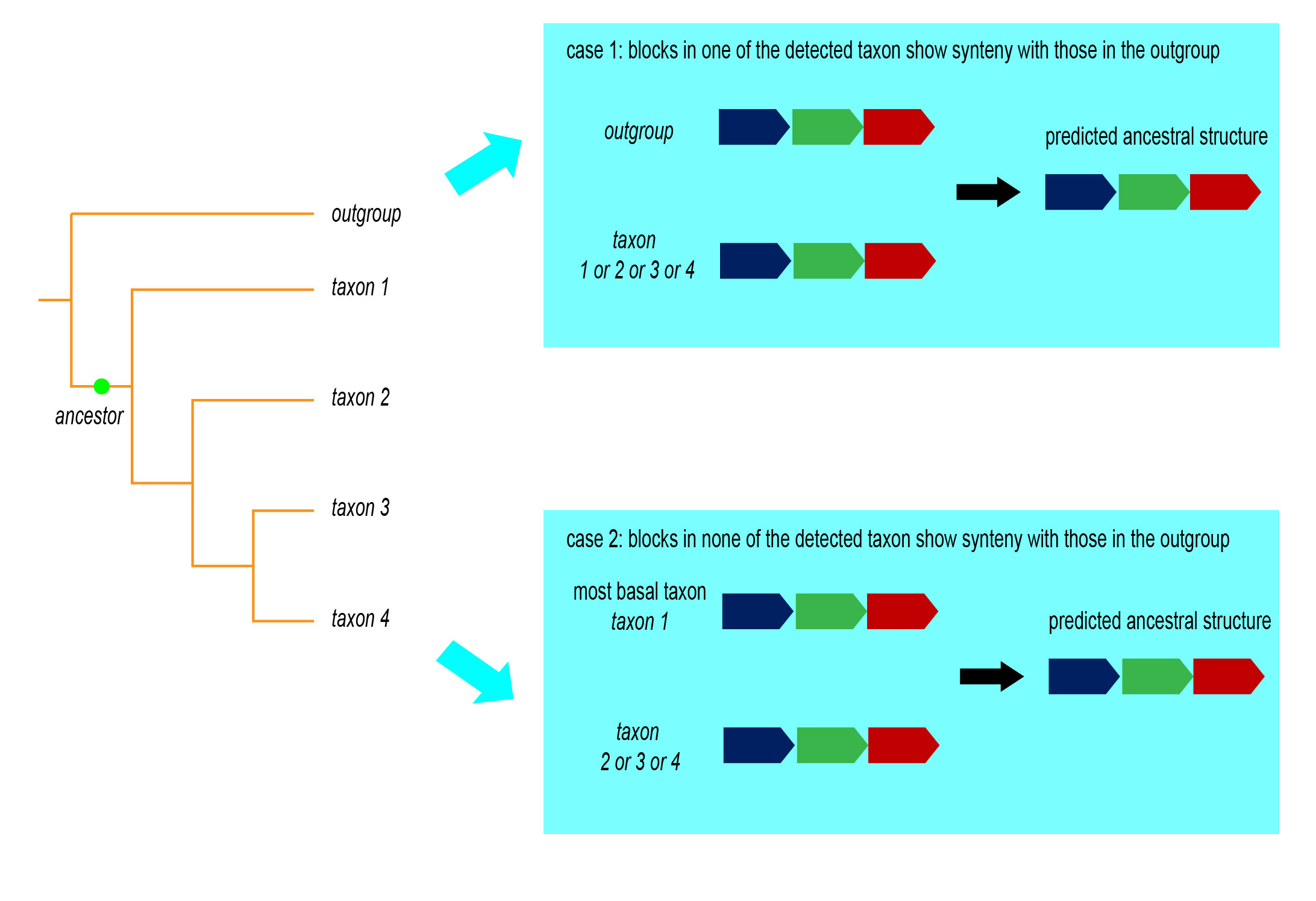

Supplement: FIGURE S1 — Algorithm of ancestral structure determination. [file Image_1.JPEG]
