# Supplementary material for: Maternal Inheritance of U’s Triangle and Evolutionary Process of Brassica Mitochondrial Genomes
Source: Front Plant Sci. 2020 Jun 12;11:805. doi: 10.3389/fpls.2020.00805 (PMC7303332; doi:10.3389/fpls.2020.00805)
Supplement: TABLE S1 — List of 12 Brassicales taxa with the mitochondrial genomes used in this study. [file Table_1.DOCX]

**Table S1**. List of 12 Brassicales taxa with the mitochondrial genomes used in this study.

| Taxon | Reference | Accession No. |
| --- | --- | --- |
| *Carica papaya* | No | NC_012116 |
| *Batis maritima* | (Grewe et al. 2014) | NC_024429 |
| *Arabidopsis thaliana* | (Unseld et al. 1997) | NC_037304 |
| *Brassica napus* | (Handa 2003) | NC_008285 |
| *Brassica carinata* | (Chang et al. 2011) | NC_016120 |
| *Brassica juncea* | (Chang et al. 2011) | NC_016123 |
| *Brassica oleracea* | (Chang et al. 2011) | NC_016118 |
| *Brassica rapa* | (Chang et al. 2011) | NC_016125 |
| *Sinapis arvensis* | (Sang et al. 2020) | NC_031896 |
| *Brassica nigra* | (Yamagishi et al. 2014) | NC_029182 |
| *Raphanus sativus* | (Tanaka et al. 2012) | NC_018551 |

**References**

Chang SX, Yang TT, Du TQ, Huang YJ, Chen JM, Yan JY, He JB, Guan RZ (2011) Mitochondrial genome sequencing helps show the evolutionary mechanism of mitochondrial genome formation in Brassica. Bmc Genomics 12. doi:Artn 497

10.1186/1471-2164-12-497

Grewe F, Edger PP, Keren I, Sultan L, Pires JC, Ostersetzer-Biran O, Mower JP (2014) Comparative analysis of 11 Brassicales mitochondrial genomes and the mitochondrial transcriptome of Brassica oleracea. Mitochondrion 19:135-143. doi:10.1016/j.mito.2014.05.008

Handa H (2003) The complete nucleotide sequence and RNA editing content of the mitochondrial genome of rapeseed (Brassica napus L.): comparative analysis of the mitochondrial genomes of rapeseed and Arabidopsis thaliana. Nucleic Acids Res 31 (20):5907-5916. doi:10.1093/nar/gkg795

Sang S, Cheng H, Mei D, Fu L, Wang H, Liu J, Wang W, Zaman QU, Liu K, Hu Q (2020) Complete organelle genomes of Sinapis arvensis and their evolutionary implications. The Crop Journal. doi:<https://doi.org/10.1016/j.cj.2019.12.001>

Tanaka Y, Tsuda M, Yasumoto K, Yamagishi H, Terachi T (2012) A complete mitochondrial genome sequence of Ogura-type male-sterile cytoplasm and its comparative analysis with that of normal cytoplasm in radish (Raphanus sativus L.). Bmc Genomics 13. doi:Artn 352

10.1186/1471-2164-13-352

Unseld M, Marienfeld JR, Brandt P, Brennicke A (1997) The mitochondrial genome of Arabidopsis thaliana contains 57 genes in 366,924 nucleotides. Nat Genet 15 (1):57-61. doi:DOI 10.1038/ng0197-57

Yamagishi H, Tanaka Y, Terachi T (2014) Complete mitochondrial genome sequence of black mustard (Brassica nigra; BB) and comparison with Brassica oleracea (CC) and Brassica carinata (BBCC). Genome 57 (11-12):577-582. doi:10.1139/gen-2014-0165
